# A reproducible optimisation protocol for calibrating prompt-based large language model workflows in evidence synthesis

**DOI:** 10.1016/j.mex.2026.104041

**Published:** 2026-07-12

**Authors:** Teo Susnjak

**Affiliations:** School of Mathematical and Computational Sciences, Massey University, Auckland, New Zealand

**Keywords:** Evidence synthesis automation, Language model calibration, Prompt optimisation, Declarative programming, Systematic reviews, Reproducible workflows

## Abstract

This methods article presents a reproducible workflow for optimising and testing improving large language models (LLMs) prompts in evidence-synthesis tasks with defined inputs and outputs. The method separates the fixed rules that define the scientific task from the editable prompt instructions that frame and apply them. It optimises those instructions against labelled or reference examples and an explicit task metric, then saves the final workflow with its specification, metric, settings, and evaluation traces so others can inspect and reuse it. The example code uses DSPy and GEPA tools, but the same logic can transfer to other prompt-optimisation frameworks that support structured task definitions, metric-guided search, and reusable outputs. Title and abstract screening is the illustrative validation case here as it provides labelled benchmark data and clear evaluation metrics. The demonstrated workflow uses a smaller *student* LLM for the scientific task and a larger *reflection* LLM to steer prompt optimisation during calibration. This work shows compilation, saving and reloading, and how optimisation budget affects a smaller student model.

• Separate *what* the model must decide (the fixed rules), from *how* it is prompted to decide (the mutable prompt instructions).
• Optimise the prompt automatically against labelled examples and a declared scoring function, rather than iterating manually.
• Package the calibrated prompt with its rules, metric, settings, traces, and evaluation logs for inspection and reuse.

## Graphical abstract

**Figure:**
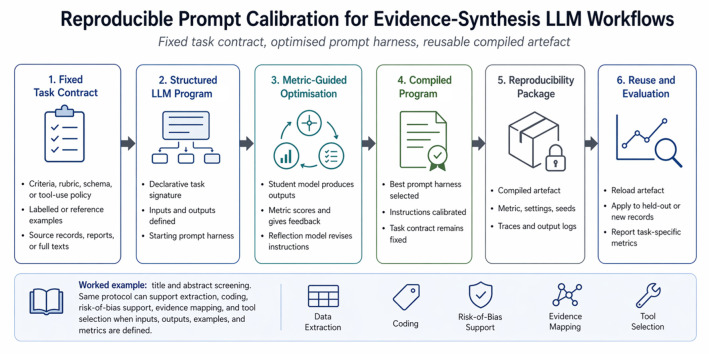

## Background

Large language models (LLMs) can help automate parts of systematic literature reviews (SLRs) and meta analyses [4]. Recent studies show that these models can support screening [5], data extraction [6], quality assessment [7], and synthesis tasks [8]. Currently, this use has a practical limit. Performance depends not only on the task and model, but also on prompt wording, model family, output-generation settings (e.g., temperature and top-p parameters), and the manual prompt-tweaking choices made during iterative development. Studies show that prompt sensitivity varies across datasets and models. Larger models are often more robust, but pockets of variability persist even in stronger instruction-tuned systems [9], [10], [11]. Repeated prompting is methodologically important because LLM outputs are probabilistic and stability should be measured rather than assumed [12]. These sensitivities matter most when researchers use smaller or locally hosted models because these LLMs tend to be cheaper and more privacy-preserving for sensitive domains. The consequence is that these models often need testing and prompt adjustment before they can be used reliably in evidence-synthesis workflows. In these cases, principled calibration can make LLM-based evidence-synthesis workflows more reliable, repeatable, and crucially for this domain, also auditable.

Specifications table

**Table:** 

Item	Description
Subject area	Computer Science; Information Science
More specific subject area	Evidence synthesis automation; research workflow automation; systematic review methods; language-model prompt optimisation; reproducible LLM workflows
Name of your method	Reproducible prompt calibration for structured evidence-synthesis LLM workflows
Name and reference of original method	DSPy declarative language-model programming [1], [2]; GEPA reflective prompt evolution [3]
Resource availability	Companion Colab notebook, local test script, saved artefact, and validation summaries are provided with the manuscript materials.

### Prompt variability and the need for calibration

Some researchers describe prompts as a way to program LLMs [13], but prompts are much more ambiguous than programming languages. Given that prompt performance is often contingent on numerous factors [9], [10], *prompt engineering* frequently turns into *ad hoc* manual refinement. While studies report prompt-dependent variability on models of all sizes [9], [11] and brittleness in SLR automation [4], [14], they generally do not provide reusable calibration protocols for structured evidence-synthesis tasks that preserve repeatability and reproducibility. To that end, this article treats prompt optimisation as calibration of an LLM workflow that produces research decisions. The workflow maps a structured research object, such as a title, abstract, full text, table, rubric, or tool request, to a structured output or decision. Even though larger and frontier commercial models may need less calibration, they nonetheless require an auditable task definition and demonstrable validation metric that support confidence in prompt stability and accuracy.

This methodological study makes three contributions. First, it formalises prompt optimisation as calibration of editable prompt instructions around fixed task rules and input-output requirements. Second, it shows how an executable metric can encode task requirements, output validity, and workflow trade-offs, using title and abstract screening as the worked validation case. Third, it defines what should be reported and saved, namely, the compiled program, fixed task specification, metric, optimisation configuration, environment record, traces, and prediction logs. These contributions are demonstrated with DSPy, a declarative language model programming framework [1], [2], and GEPA, a reflective prompt optimiser [3], but the protocol is intended to generalise to other prompt-optimisation frameworks with structured task definitions, executable metrics, and reusable compiled outputs. Thus, the same protocol can be implemented in other optimisation toolchains that support structured task definitions, executable metrics, metric-guided search, and reusable outputs [15].

## Method details

### Overview

The method requires four ingredients: (1) fixed task rules, which define the scientific requirements; (2) editable prompt instructions, which define how those requirements are presented to the model; (3) labelled or reference examples, which provide calibration and held-out evaluation cases; and (4) an executable metric, which scores outputs against the task policy and provides feedback for optimisation. Collectively these components define the calibration setting. The protocol optimises the editable prompt within a fixed budget and saves the calibrated workflow for reuse.

In DSPy, the calibrated workflow is represented as a *language model (LM) program*. This does not mean a conventional software program in the usual sense. It means a structured object that combines input fields, output fields, task instructions, model calls, and evaluation logic. In this work, program refers to this DSPy-style prompt-based workflow, namely, the object that GEPA calibrates and that can later be saved, reloaded, inspected, and reused. This work also does not introduce a new prompt-optimisation algorithm. Instead, it contributes a *reproducible procedure* for applying existing prompt-optimisation frameworks to structured evidence-synthesis and scientific LLM workflows. The protocol demonstrates *how to* (1) fix the scientific task rules, (2) calibrate the prompt instructions around those rules, (3) make the optimisation metric explicit, and (4) preserve the compiled program with enough information for inspection and reuse.

Title and abstract screening is a helpful illustrative use case because it is a common and well-defined evidence-synthesis task with labelled benchmark data and clear evaluation metrics. The same calibration pattern can, in principle, be adapted to other repeatable and structured evidence-synthesis tasks, including data extraction, study-characteristic coding, risk-of-bias support, evidence-domain mapping, and search-query generation, provided that the task has well-defined inputs, outputs, reference examples, and an executable metric. The protocol is less directly applicable to open-ended synthesis, exploratory hypothesis generation, or interpretive judgement tasks where no stable reference output or scoring policy can be defined. In those cases, the same packaging principles remain useful, but metric-guided prompt optimisation becomes harder to validate. Again, the critical requirement is that inputs, outputs, reference examples, and task metrics can be clearly expressed. For example, Appendix E gives an alternative schematic for a data-extraction adaptation. The accompanying Colab notebook^1^, contains the complete runnable Python source code. Meanwhile, the article body here includes the key method-defining code snippets only while small example records and notebook-specific execution cells are confined to the Colab notebook to preserve readability. The presented protocol has four steps:

1. define the task contract as a structured LM-program signature;
2. codify the task standard using labelled or reference examples and an executable metric;
3. compile the prompt harness with an optimisation framework under a fixed budget;
4. save, reload, evaluate, and package the compiled artefact.

**Fig. 1:**
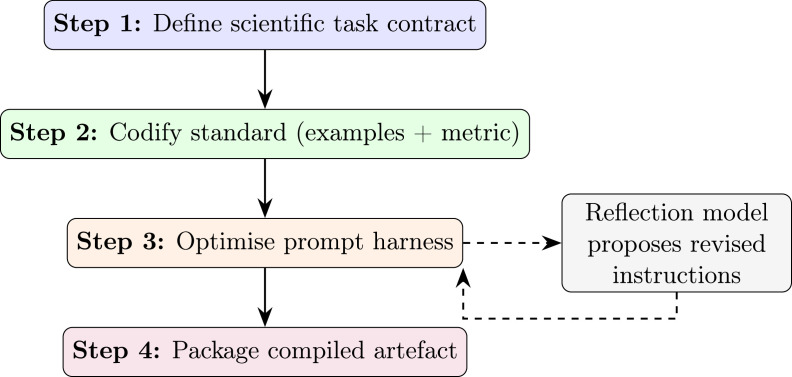
Generic calibration workflow for structured prompt-based LLM programs. The title-and-abstract screening experiment is one instantiation of this workflow. Dashed arrows show the reflective optimisation loop inside GEPA.

Fig. 1 summarises the four-step calibration workflow. Step 1 defines the scientific task contract, Step 2 turns that contract into labelled or reference examples and an executable metric, Step 3 optimises the prompt harness, and Step 4 packages the compiled artefact for inspection, reuse, and evaluation. Table 1 clarifies what is meant by “fixed” and “mutable” in this workflow, while Table 2 shows how the same calibration pattern can be adapted and is generalisable beyond title-and-abstract screening. This workflow has three parts. The first part is the scientific task contract which are the substantive rules that define the task, such as eligibility criteria, an extraction schema, a coding manual, a risk-of-bias rubric, reference labels, or a review policy. The second part is the machine-readable interface: the input fields, output fields, label set, parsing rules, and validity constraints that define what the program must receive and return during a calibration run. These first two parts remain fixed. The third part is the mutable prompt harness. This is the instructional wrapper around the fixed task contract and interface. It may include task framing, domain context, decision guidance, near-miss explanations, uncertainty handling, examples of acceptable checks, and trace-format guidance. This is the part where the prompt wording is revised, and it is the only part GEPA is permitted to change for the target LLM. In this article, the phrase “fixed task contract” thus refers to the scientific task contract and the machine-readable interface together, unless otherwise specified.

**Table 1:** Three-layer architecture separating the scientific task rules, the machine-readable interface, and the mutable prompt harness. During calibration, GEPA may revise the prompt harness but not the scientific task contract or the interface. Abstract screening is one instantiation of this pattern.

Layer	Fixed or mutable?	Examples
Scientific task contract	Fixed	Eligibility criteria, extraction schema, coding manual, reference labels, review policy
Machine-readable interface	Fixed within a calibration run	Input fields, output fields, label set, JSON or DSPy markers, parser, output-validity rules
Prompt harness	Mutable	Task wording, decision guidance, near-miss guidance, examples of checks, edge-case instructions

**Table 2:** Examples of repeatable evidence-synthesis and research-support tasks that can be expressed using the same calibration protocol. The present paper validates the protocol using title and abstract screening, but the methodological pattern applies whenever a task can be specified through structured inputs, outputs, reference examples, and an executable metric.

Task type	Fixed task contract	Mutable prompt harness	Possible metric
Title/abstract screening	Inclusion/exclusion criteria and labelled records	Screening instructions, uncertainty guidance, edge-case handling	Recall, precision, *F*_1_, workload utility
Data extraction	Extraction schema and reference extractions	Extraction order, field-use guidance, evidence-span instructions	Exact or partial field match; evidence support score
Full-text eligibility	Eligibility criteria and adjudicated decisions	Clause-by-clause assessment procedure	Agreement with reviewer labels; false-exclusion penalty
Risk-of-bias support	Assessment rubric and expert ratings	Domain-specific rating instructions and justification format	Weighted agreement; class-pair cost matrix
Study-characteristic coding	Coding manual and labelled examples	Category boundary guidance and examples	Macro-*F*_1_; class-balanced agreement
Evidence-domain mapping	Domain taxonomy and validated mappings	Taxonomy application procedure	Multiclass agreement; confusion-cost utility
Search-query generation	Search objective and known relevant studies	Query-construction and expansion instructions	Recall of known studies; query precision proxy
Tool selection or workflow routing	Available tools, routing policy, and reference decisions	Tool-choice instructions and failure-handling rules	Tool-choice accuracy; cost-weighted routing utility

### Materials and resources

The method shown uses Python, a prompt-optimisation framework, labelled or reference examples, a *student* language model for task execution, and a *reflection* language model for prompt revision. The student model is the LLM of interest and is the target model which is to be deployed after the prompt calibration. In settings with cost, privacy, or governance constraints, this will usually be the smaller, less expensive or locally hosted model. The reflection model can be more capable and expensive because it is used only during compilation, and its role is significantly less token-intensive. It inspects failures, reads metric feedback, and proposes revised instructions for the student program. This separation allows researchers to use a stronger model during calibration without using it for routine task execution. Recent literature shows that calibrated smaller models can, in some settings, even approach larger-model performance at substantially lower cost [3], [16].

Temperature settings control the two roles differently. The student model is run with temperature =0.0 to reduce avoidable variation during evaluation and execution of the scientific task. The reflection model is run with a higher temperature, typically around temperature=1.0, to generate varied prompt revisions during search. The Colab notebook uses example provider strings only and comparable smaller models of choice can be substituted for the student and stronger models can likewise be substituted for reflection^2^ The Colab notebook embeds a 12-record demonstration dataset with explicit train, val, and test splits to keep the method visible. A separate validation experiment tests the end-to-end workflow on non-toy data. The tutorial code was tested with the package versions reported in Appendix D. GEPA is an active optimisation component in the DSPy ecosystem, and its API or serialisation behaviour may change across package releases. Readers should therefore use the recorded package versions when reproducing the tutorial exactly, and should treat newer versions as requiring re-verification of the notebook, compiled artefact, and evaluation outputs.

### Step 1: Define the task contract

The DSPy signature turns the task rules into input and output fields. It states what information the model receives and what fields it must return. In different evidence-synthesis workflows, this contract may describe a screening decision (i.e., include or exclude), an extraction schema, a coding rubric, a risk-of-bias judgement, or a tool-selection policy. It also contains the starting prompt harness, which is a concise description with relevant task context, scope, reasoning or extraction procedure, and guidance for satisfying the fixed output contract. This component can be regarded as *context engineering*. This prompt-design and enrichment step matters most for smaller student models. A thin instruction gives GEPA little material to vary so it can propose a meaningful alternative prompt, while a richer harness gives the reflection model more structure to inspect and work with when examples fail. Useful context can include task scope, study aims, near-miss cases, expected evidence use, and the required form of the output.

Listing 1 mirrors the Colab notebook and gives the screening instantiation used in the worked example. The same pattern can be rewritten for other tasks. For data extraction, the input fields might include the full text and extraction schema, while the output fields might include structured study characteristics, effect sizes, populations, interventions, comparators, outcomes, and evidence spans. For tool selection, the input fields might include the task request and available tools, while the output fields might include the selected tool, arguments, and confidence or fallback policy. In the screening instantiation, the inclusion and exclusion criteria are supplied through the criteria input rather than being embedded as mutable prompt text. This separation is deliberate. The criteria represent the scientific decision rule agreed for the review, and GEPA should not rewrite/mutate them. GEPA is however allowed to revise the prompt harness around those criteria so that the student model applies them more reliably. Once defined, the criteria are documented and kept unchanged during calibration.

**Listing 1:**
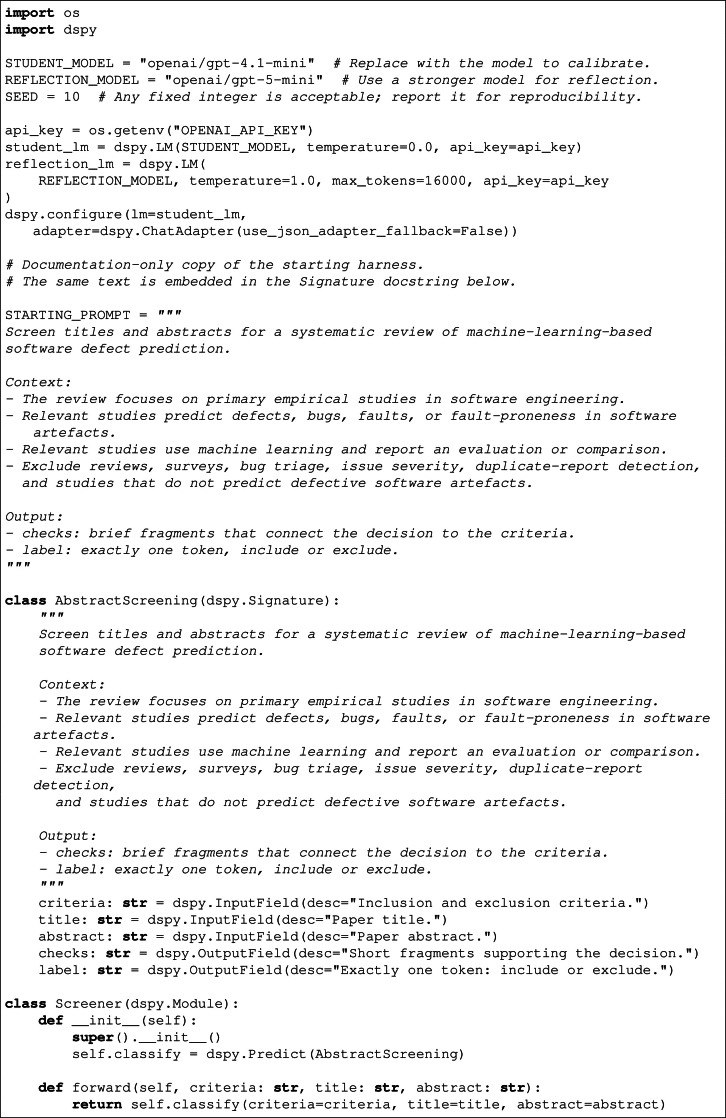
DSPy task contract for title and abstract screening.

For readability, STARTING_PROMPT is shown as the conceptual prompt harness; in the DSPy implementation below, the same text is embedded in the AbstractScreening signature docstring. In the validation experiment as well as in the Colab notebook, the review criteria were extracted from the full text of the systematic review [17] (referred to as Study 41), which is the secondary literature review study identified as reference [41] in the SESR-Eval [18] dataset; the frozen criteria block used in the validation comparison is reported in the Appendix Listing A.6.

### Step 2: Define examples and the scoring metric

Similar to machine learning, GEPA optimises against examples and a metric rather than against instructions alone. The example dataset is split into training examples that teach the task, validation examples that guide GEPA in optimising the prompts, and held-out test examples that provide a small feasibility check.

The metric is task-specific because different evidence-synthesis tasks have different error costs. In the general protocol, the metric defines what the optimiser should reward, what output formats are valid, and how competing errors should be balanced. For data extraction, this may mean combining exact field matches, partial credit for near-correct values, evidence-span checks, and penalties for values that are not supported by the text. For risk-of-bias support, it may mean weighting disagreements so that adjacent-category errors are treated as less serious than errors at opposite ends of the rating scale. For tool-selection tasks, it may mean rewarding the correct tool and valid arguments while penalising unsafe or unnecessary tool calls. For screening, the main trade-off is between false exclusions and false inclusions. Listing 2 therefore shows one concrete metric for binary screening, where false exclusions are penalised more strongly than false inclusions.

**Listing 2:**
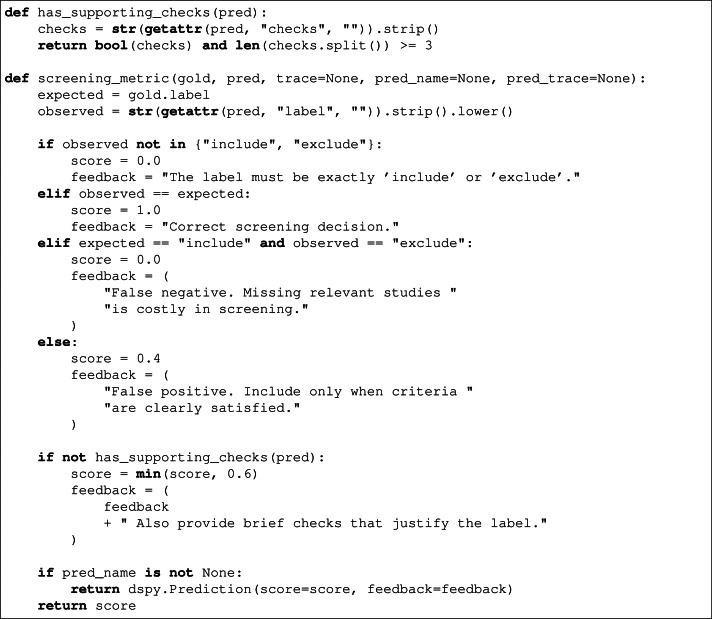
Compact GEPA metric used in the Colab notebook.

The metric is the main place where researchers define what counts as a good output. A standard accuracy metric tells the optimiser whether an output was correct, but it does not explain how the output failed or how severe that failure is in the downstream workflow. GEPA can use richer feedback: the metric can return both a numeric score and a short natural-language diagnosis. This feedback gives the reflection model information it can convert into better instructions/prompts. For abstract screening, the metric should encode the review policy. Given that missing a relevant study is usually worse than sending an irrelevant study to full-text review, the example metric here (Listing 2 shows the compact metric used in the Colab notebook)^3^ penalises false exclusions more strongly than false inclusions. It also checks for a minimal justification in the checks field. This constraint discourages prompt revisions that obtain the right label while dropping the justification needed to inspect the decision later.

Researchers can change the metric to match the task. They can adjust the false-positive score, add penalties for malformed output, require criterion-level justifications, or add task-specific constraints such as “route uncertain records to human review”. The metric should be fixed before running the final optimisation and reported with the compiled artefact.

#### Designing the GEPA metric as a task-specific scoring function

The compact metric in Listing 2 implements the error-cost policy for binary screening. A plain exact-match objective would assign full credit to a correct label and zero to any error. That is too coarse for systematic review workflows, because a false negative removes a potentially relevant study from the pipeline, whereas a false positive mainly increases downstream screening workload. This asymmetry is well established in screening research [19]. Table 3 shows the scoring used in this example and how it can be adjusted for similar use cases.

**Table 3:** Asymmetric scoring for binary screening. The false-positive value (0.4) is a policy choice: it treats a false inclusion as clearly inferior to a correct decision while distinguishing it from a false exclusion, which is the more severe screening failure.

Gold label	Predicted label	Error type	Screening meaning	Score
include	include	correct	relevant study retained	1.0
exclude	exclude	correct	irrelevant study removed	1.0
include	exclude	false negative	relevant study missed	0.0
exclude	include	false positive	extra full-text workload	0.4

The false-positive score of 0.4 is a policy parameter rather than a default value for all screening tasks. Increasing this value makes the optimiser more tolerant of over-inclusion and can shift the calibrated program towards higher recall and higher downstream full-text screening workload. Decreasing it increases pressure for precision, but may raise the risk of false exclusions. Users applying this protocol to a new review should therefore report the chosen value and, where possible, perform a sensitivity analysis over plausible values such as 0.2, 0.4, and 0.6. The present validation uses 0.4 as a moderate partial-credit setting to demonstrate the protocol, not as a recommended default for all evidence-synthesis tasks.

The GEPA metric should guide optimisation, not replace final held-out test metrics. During optimisation, it combines label correctness, asymmetric error costs, output-validity checks, and textual feedback for reflection. After optimisation, performance should still be reported on held-out records using conventional measures such as accuracy, precision, recall, and *F*_1_. Appendix B provides the component-level details, shows how label normalisation and trace validation were implemented, and discusses how this design extends to multiclass screening.

#### Why GEPA rather than few-shot optimisation?

GEPA is used here because the protocol requires revising prompt instructions using detailed metric feedback, not only selection or bootstrapping of few-shot examples. Few-shot optimisers are useful when the main task is to choose demonstrations for inclusion in a prompt. Structured evidence-synthesis tasks often fail at boundary conditions where a model may include a near-miss study, over-apply an exclusion criterion, omit an evidence field, or choose the wrong workflow tool. These failures are better addressed by revising task instructions from case-grounded metric feedback while preserving the fixed task contract and machine-readable interface. Table 4 situates GEPA among common alternatives.

**Table 4:** Comparison of prompt-calibration and model-adaptation strategies relevant to structured evidence-synthesis workflows.

Approach	What it optimises	Reflection model?	Best suited for	Limitation
Manual prompt revision	Human-written instructions	No	Small exploratory tasks	Poor audit trail; low reproducibility
Few-shot bootstrapping	Demonstration selection	No or optional	Tasks where examples drive behaviour	Less direct instruction repair
GEPA	Instruction harness using metric feedback	Yes	Boundary cases, structured outputs, task-policy feedback	Higher calibration cost
Manual reflection loop	Human- or LLM-generated revisions	Optional	Framework-agnostic settings	Less standardised packaging
Fine-tuning	Model weights	No at inference	Large labelled datasets	Higher setup cost; less prompt-level inspectability

### Step 3: Compile the program with GEPA

With the task contract, examples, and metric fixed, the next step is to compile the unoptimised Screener. Listing 3 shows the minimal GEPA compilation call used in the Colab tutorial.

**Listing 3:**
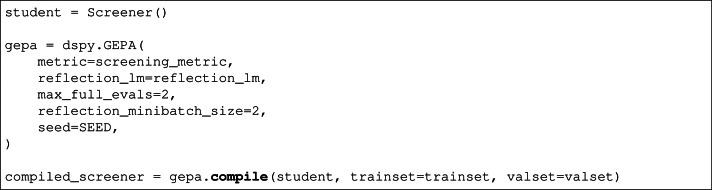
Bounded GEPA compilation step.

The main budget parameter is max_full_evals. It controls how many candidate program variants GEPA can evaluate before returning the revised DSPy workflow. Larger values give GEPA more opportunity to improve the prompt harness, but they increase the cost and may fit too closely to the validation examples when the validation set is small. The example uses max_full_evals=2 only to keep the Colab run inexpensive. The validation experiment varies this parameter, reported as max_eval, to examine the cost-performance trade-off.

reflection_minibatch_size controls how many examples are shown to the reflection model when proposing revised instructions. Small minibatches are cheap and expose local errors; larger minibatches can reveal broader failure patterns, but may mix unrelated cases and increase cost. For small tutorials, values of 2 or 3 are sufficient. For real evidence-synthesis tasks, this value should be increased only when enough diverse labelled or reference examples are available.

Other parameters are implementation controls. num_threads affects evaluation speed, track_stats supports debugging and trace reporting, and skip_perfect_score controls whether already-correct examples are omitted from reflection. Table 5 summarises the parameters readers need to understand first. After compilation, the revised instruction text can be inspected and saved as part of the compiled DSPy artefact rather than treated as an untracked manual prompt change. Appendix A shows the relationship between the starting harness and a compiled harness.

**Table 5:** Key GEPA parameters and practical guidance for tuning.

Parameter	Controls	Increase when	Risk
max_full_evals	Search budget	Held-out performance remains below the desired performance level and validation examples are sufficiently diverse	Over-specialisation
reflection_minibatch_size	Examples shown to reflector	Failures are diverse	Diluted feedback
num_threads	Parallel evaluation	Runtime too long	Rate limits
temperature (reflection)	Revision diversity	Revisions too similar	Unstable runs

#### Cost and budget reporting

max_full_evals should be reported as both an optimisation hyperparameter and a main factor affecting cost. GEPA cost depends on student-model evaluations, reflection-model calls, validation-set size, minibatch size, maximum reflection tokens, provider pricing, and retry behaviour. When available, users should record model calls, input tokens, output tokens, wall-clock time, and provider cost. When token accounting is unavailable, they should at least report the GEPA budget, validation-set size, reflection minibatch size, model identifiers, temperatures, seeds, and execution date. Calibration cost is usually one-off or occasional, so it should be interpreted relative to the number of future records, reviews, or reruns over which the compiled artefact will be reused. Table 6 reports the budget and model settings available for this study.

**Table 6:** Budget and model settings for Colab notebook verification and archived validation. The tutorial used reflection_minibatch_size=2; the validation ablation used reflection_minibatch_size=8. Exact token-level cost was not available from the local execution logs. Future deployments should log model calls, input tokens, output tokens, wall-clock time, provider cost, model identifiers, temperatures, execution date, reflection_minibatch_size, and max_full_evals.

Setting	Student model	Reflection model	max_full_evals	Seeds
Tutorial smoke test	openai/gpt-4.1-mini	openai/gpt-5-mini	1	10
Validation ablation	openrouter/qwen/ qwen-2.5-7b-instruct	openai/ gpt-5-mini	2, 6, 12, 24	10, 15, 25, 35, 42

### Step 4: Package and reuse the artefact

Next, the compiled program should be saved and reloaded before being used in a study, so the optimised prompt can be reused and checked later. Listing 4 evaluates the compiled program, saves it, reloads it into a fresh Screener, and verifies that the reloaded artefact produces the same held-out predictions.

**Listing 4:**
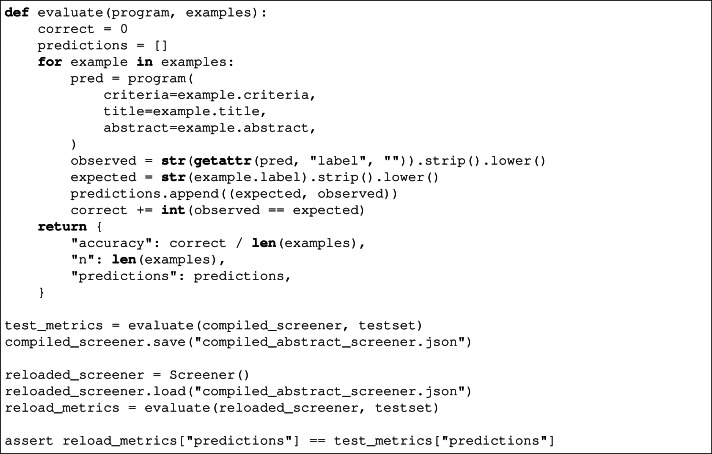
Evaluation and artefact round trip.

The Colab notebook uses this round-trip check only to verify artefact reuse^4^. The JSON save used here stores the program state needed to reload the same program architecture, including the compiled signature or instruction state and any stored predictor state such as demonstrations or model assignments. It does not by itself preserve the full research provenance needed to interpret or reproduce the calibration. That provenance must be recorded separately, including the dataset version, split identifiers, metric source, optimisation configuration, model and provider details, package versions, seeds, adapter settings, and evaluation or prediction logs. DSPy also supports whole-program saving with save_program=True, but that option uses cloudpickle and still does not replace the need to document the dataset, metric, splits, provider-side model version, optimisation configuration, and evaluation logs.

### Transparency and inspection of saved outputs

A central aim of the protocol is to ensure that prompt calibration is saved and reviewable rather than lost after experimentation. In ordinary prompt engineering, the final prompt is often the only visible object, while failed variants, metric assumptions, data splits, and output-normalisation decisions are often lost. In this workflow, the compiled LLM workflow is treated as a saved research object with provenance. The saved artefact should therefore be accompanied by the fixed task specification, starting signature, optimisation metric, GEPA configuration, random seeds, split identifiers, compiled prompt harness, software environment, and held-out prediction or output logs. This packaging supports two forms of reproducibility. The first is execution-level reuse: the saved program can be reloaded and evaluated again under recorded conditions. The second is clear reporting of the task rules, metric, prompt text, traces, and evaluation outputs, so readers can inspect why a calibration was selected and what trade-offs it encodes. The artefact should therefore be treated as a saved and reviewable research object, not as a guarantee of identical results across model providers or later model versions.

### Tuning and troubleshooting guidance

Users should adjust the protocol in the following order: (1) check data and label consistency; (2) refine the metric so that feedback identifies specific failure types; (3) increase max_full_evals; and (4) adjust reflection_minibatch_size. Table 7 lists common failure modes and first remedies.

**Table 7:** Common failure modes and recommended first remedies.

Symptom	Likely cause	First remedy
Invalid outputs	Weak output contract	Tighten label field description; add metric penalty for out-of-schema labels
Over-selective decisions	Recall pressure too weak; false negatives under-penalised	Strengthen false-negative feedback; add examples where relevant studies are easy to miss
Over-permissive decisions	False-positive score too generous; inclusion evidence too weak	Lower false-positive partial credit; require explicit evidence for inclusion criteria
Validation rises, test drops	Over-specialisation	Reduce budget; increase validation diversity; simplify the metric
Trace missing or malformed	Output trace not rewarded	Cap score for missing checks; require criterion-level fragments

### Minimal replication recipe

The following steps summarise the complete protocol. Each step corresponds to a section above and a code in the companion notebook.

1. Install the package versions recorded in Appendix D.
2. Load source records or documents for the target evidence-synthesis task.
3. Finalise the criteria, rubric, extraction schema, coding manual, or tool policy before optimisation.
4. Define a DSPy Signature that declares inputs, outputs, and the initial prompt template.
5. Build dspy.Example objects with explicit train/validation/test splits.
6. Define the task metric with output-validity checks, feedback, and task-specific error weighting.
7. Run GEPA with a fixed seed and bounded budget, such as max_full_evals.
8. Save the compiled program to a JSON artefact.
9. Reload the artefact into a fresh program object and verify matching outputs.
10. Report task-specific held-out metrics, such as accuracy, precision, recall, *F*_1_, extraction agreement, field-level match, weighted agreement, or routing accuracy.

## Method validation

This validation checks whether the protocol can show how trade-offs change when the task rules are fixed. It is not intended to rank models or to make a general performance claim about GEPA. It demonstrates three points: (1) a smaller student model can be calibrated under a transparent protocol, (2) optimisation budget can change the resulting performance trade-off, and (3) the compiled artefact can be evaluated and reused with documented provenance.^5^

The validation used a binary title-and-abstract screening task with Qwen-2.5-7B-instruct as the student model and GPT-5-nano as the reflection model, applied tp the SESR-Eval Study 41 as the benchmark case. Study 41 contains 1194 candidate records for the secondary review *Machine learning in software defect prediction: A business-driven systematic mapping study* [17], and is one of 24 software-engineering secondary-study screening datasets in SESR-Eval [18]. The inclusion and exclusion criteria were extracted from the Study 41 source article, reviewed as a compact task contract, and frozen before calibration. Across five random seeds, stratified train/validation/test splits were generated under the same fixed criteria.

The principal comparator was the structured unoptimised DSPy program, evaluated before calling gepa.compile(). It used the same criteria, title, and abstract inputs, the same checks and label outputs, and the same student model, temperature, adapter configuration, label normalisation, held-out examples, and confusion-matrix evaluation as the GEPA conditions. The before-and-after differences therefore measure the effect of GEPA on the same structured program. Where a label-only direct prompt is reported, it is only a practical reference for ordinary direct prompting, not the principal comparator for attributing the effect of GEPA calibration. Appendix A defines the structured baseline. The validation varied only the GEPA budget. The tested max_full_evals values were 2, 6, 12, and 24, reported as max_eval. The metric, model, data splits, and evaluation procedure were held constant. Performance was computed on the held-out test set using confusion-matrix counts and is reported as mean  ±  sample standard deviation across five runs.

**Fig. 2:**
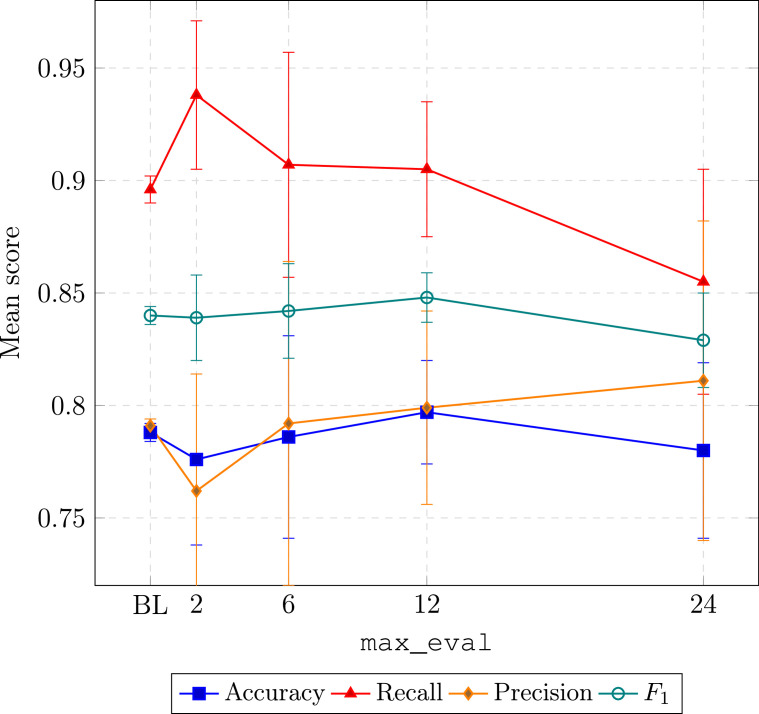
Budget-sensitive performance trade-offs across GEPA settings. BL denotes the structured unoptimised baseline. Points show means across five runs; error bars show  ± 1 sample standard deviation. The figure shows that increasing GEPA budget did not produce steady improvement in this validation: low budget favoured recall, a moderate budget gave the best balanced mean performance, and the largest tested budget reduced mean *F*_1_ and utility.

The observed shifts were modest and should be read as changes specific to this validation, not as proof that GEPA always improves performance. The structured baseline achieved accuracy 0.788 ± 0.004 and $F_{1}=0.840\pm 0.004$, with recall 0.896 ± 0.006 and precision 0.791 ± 0.003 for the include class. A low GEPA budget (max_eval=2) produced the highest recall (0.938 ± 0.033) but increased the predicted include rate, implying more downstream full-text screening work. The most balanced setting in this validation was max_eval=12, with accuracy 0.797 ± 0.023, $F_{1}=0.848\pm 0.011$, and label-level utility 0.855 ± 0.007. Relative to the structured baseline, this setting had $\Delta F_{1}=+0.008\pm 0.008$ and Δutility =+0.008±0.005, while changing the predicted include rate by only +0.002±0.061. A larger budget (max_eval=24) produced lower mean *F*_1_ and utility in this validation setting, illustrating why optimisation budget should be treated as a hyperparameter rather than a setting that always helps when increased. Fig. 2 visualises these trade-offs; full replication settings, metric tables, paired deltas, and SESR-Eval contextual comparisons are provided in Appendix C.

## Limitations

This method calibrates a model to apply documented task rules; it does not replace expert judgement in evidence synthesis. Borderline eligibility decisions, ambiguous extracted fields, risk-of-bias judgements, and final synthesis decisions should remain under human oversight. Performance depends on the quality and representativeness of the labelled or reference examples, the stability of the selected model provider, and how well the metric reflects the task rules. Very small validation sets can also lead GEPA to over-specialise to local examples, especially when max_full_evals is increased without more diverse validation data.

The validation is limited to one binary screening task, one benchmark case, one student model, and a small number of repeated runs. The paired GEPA deltas should therefore be read as performance trade-offs under fixed task rules, not as a general claim that GEPA will improve all evidence-synthesis tasks or all models. Other tasks, including extraction, coding, risk-of-bias support, and tool routing, require task-specific signatures, reference examples, and metrics, although the same steps for calibration, saving, and reuse still apply. Exact numerical reproduction is not guaranteed across model-provider changes. API adapters, serving infrastructure, tokenizer behaviour, and provider-side model versions can change, so reproduction may require pinning the model identifier, SDK, adapter versions, package versions, and execution environment recorded in Appendix D.

## Ethics statements

This study used public benchmark records and published secondary-study materials, and did not involve human participants, private personal data, intervention, or recruitment. Institutional ethics approval was therefore not required.

## Declaration of generative AI and AI-assisted technologies in the manuscript preparation process

During the preparation of this work, the author used generative AI and AI-assisted tools to support manuscript drafting, language refinement, brainstorming, code checking, and verification of selected implementation details. After using these tools, the author reviewed, edited, and verified the content as needed and takes full responsibility for the final manuscript, code, analyses, and conclusions.

## Funding

This research did not receive any specific grant from funding agencies in the public, commercial, or not-for-profit sectors.

## Supplementary material and/or additional information

Supplementary materials include the companion Colab notebook, the local smoke-test script, the saved compiled-program artefact generated by the tutorial, and the small example dataset embedded in the notebook. The larger validation uses SESR-Eval Study 41 records and the Study 41 source article cited above. Table 8 provides a compact checklist of reported reproducibility items and their locations. The Colab notebook was executed end-to-end in a clean runtime on 24 April 2026 using the package versions reported in Appendix D. The saved artefact reloaded successfully and produced the same basic test predictions and metrics as the compiled programme in the Colab run.

**Table 8:** Reproducibility checklist for the validation experiment.

Item	Reported?	Location
Model identifiers (student + reflection)	Yes	Listing 1, Appendix D
Random seeds	Yes	Table C.11
Train/validation/test split sizes	Yes	Table C.11
Structured baseline, direct-prompt reference, and frozen criteria	Yes	Appendices A and A
Metric source code	Yes	Listing 2, Appendix B
Execution environment	Yes	Appendix D
Saved compiled artefact	Yes	Supplementary material
Evaluation metrics reported	Yes	Tables C.12–C.13

## Declaration of competing interest

The author declares that he has no known competing financial interests or personal relationships that could have appeared to influence the work reported in this paper.

## Figures and Tables

**Listing A.5:**
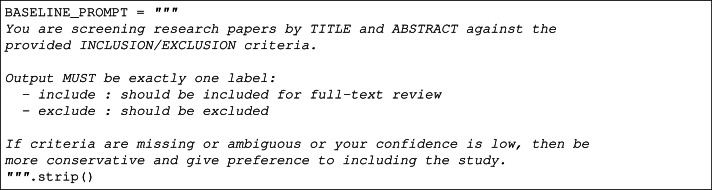
Label-only direct-prompt reference.

**Listing A.6:**
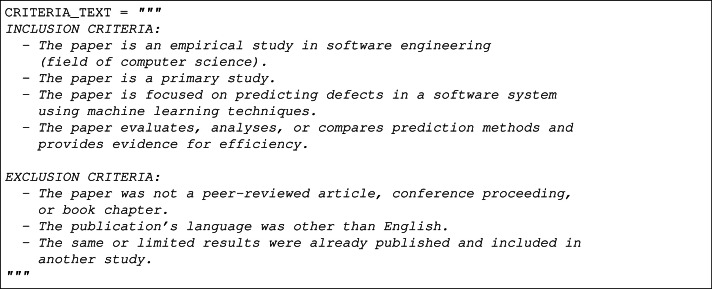
Frozen criteria block used in the validation comparison.

**Listing A.7:**
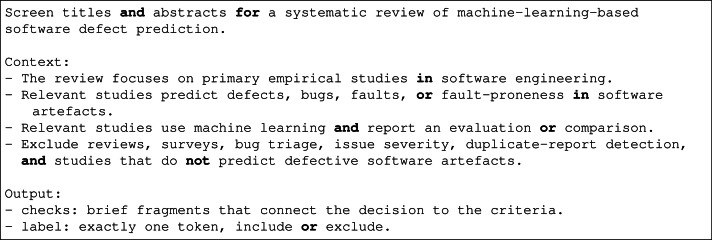
Starting prompt harness used in the Colab notebook and as the mutable starting point for GEPA.

**Listing A.8:**
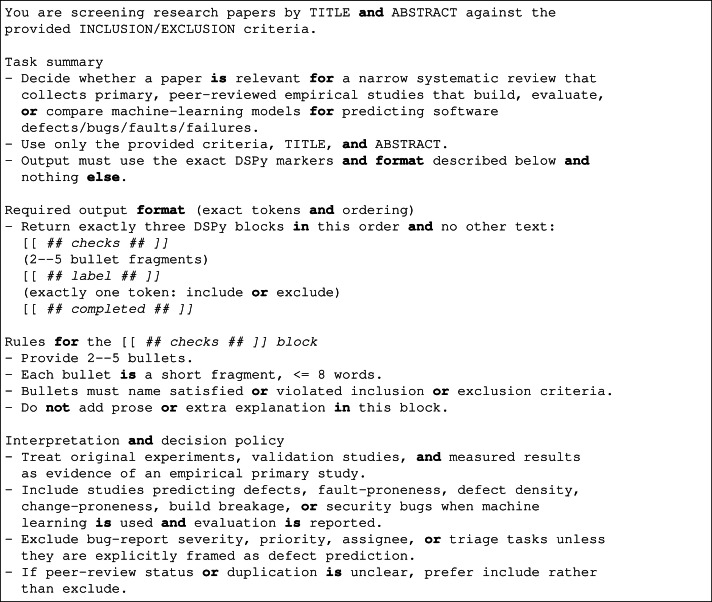
Excerpt from an optimised screening harness produced by GEPA.

**Listing B.9:**
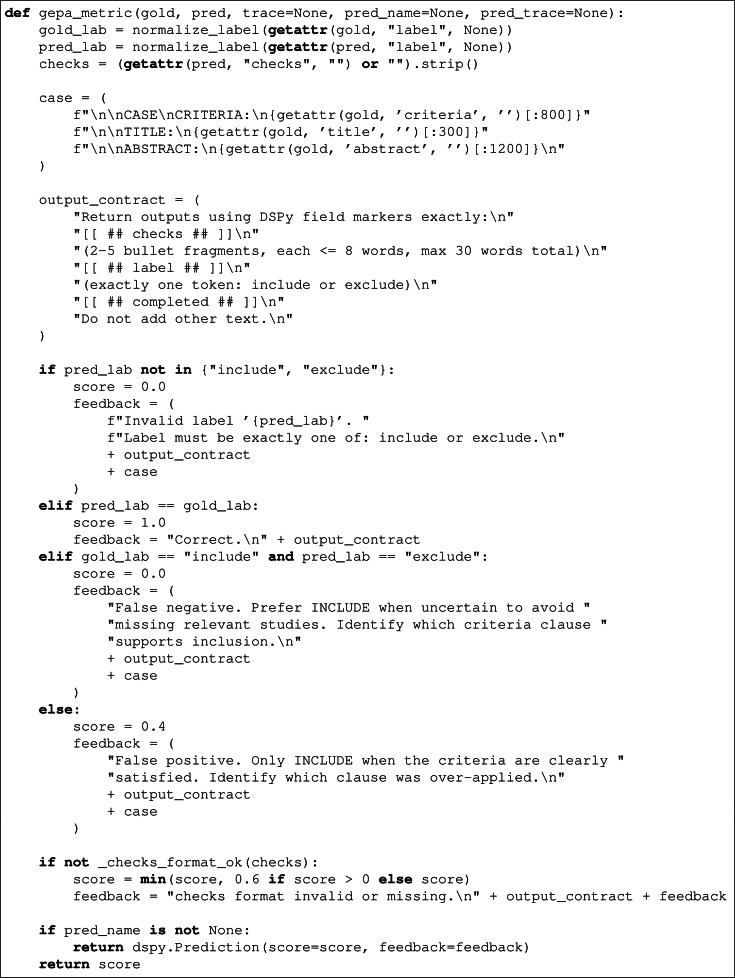
Expanded local-testing metric with case-grounded feedback.

**Listing B.10:**
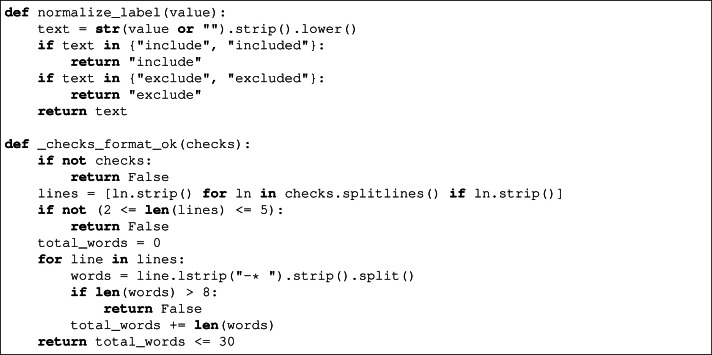
Validation helper utilities used by the expanded metric.

**Table B1:** Interpretation of false-positive score and checks cap settings.

Parameter	Setting	Interpretation
False-positive score	Lower, e.g., 0.1–0.3	Stricter inclusion, with higher precision pressure.
False-positive score	Moderate, e.g., 0.4–0.6	High recall with some penalty for false positives.
False-positive score	Higher, e.g., 0.7–0.9	Strong tolerance for over-inclusion, supporting a recall-prioritising workflow.
Checks cap	Lower	Stronger enforcement of the audit trace.
Checks cap	Higher	Label correctness dominates, while trace compliance is weaker.

**Table B2:** Post-hoc label-level utility under alternative false-positive scores, computed from the archived confusion matrices. Values are mean ± sample SD across five runs.

Condition	FP score 0.2	FP score 0.4	FP score 0.6
Structured baseline	0.818 ± 0.004	0.847 ± 0.004	0.877 ± 0.004
GEPA max_eval=2	0.813 ± 0.027	0.850 ± 0.017	0.887 ± 0.009
GEPA max_eval=6	0.817 ± 0.030	0.849 ± 0.016	0.880 ± 0.006
GEPA max_eval=12	0.826 ± 0.015	0.855 ± 0.007	0.884 ± 0.004
GEPA max_eval=24	0.806 ± 0.028	0.832 ± 0.019	0.858 ± 0.016

**Table C1:** Minimum replication configuration for the validation experiment.

Component	Value	Why it matters
Dataset subset	SESR-Eval Study 41	Defines task population
Criteria source	Study 41 full text	Defines screening protocol
Criteria extraction	GPT-5 Nano, reviewed/frozen	Separates extraction from optimisation
Student model	Qwen-2.5-7B-instruct	Student model being calibrated
Reflection model	GPT-5-mini	Prompt revision model (calibration only)
Train/val/test sizes	59/59/1076 for the structured baseline and GEPA runs	Defines labelled budget and held-out evaluation set
Seeds	10, 15, 25, 35, 42	Enables repeated-run replication
GEPA budgets tested	2, 6, 12, 24	Defines optimisation effort
Metric	Asymmetric utility + checks penalty	Defines optimisation objective
Student temperature	0.0	Controls stochasticity

**Table C2:** Qwen-2.5-7B abstract screening results (mean ± sample SD over 5 random splits; positive class is include). $\hat{\pi }\left(\text{include}\right)=\left(TP+FP\right)/N$ approximates downstream full-text screening workload. Utility is the label-level asymmetric utility corresponding to the GEPA cost shaping, U=(TP+TN+0.4FP)/N. It excludes auxiliary penalties for malformed checks or invalid field formatting, which are used during optimisation but are not represented in the confusion matrix. MCC is Matthews correlation coefficient and κ is Cohen’s kappa computed from the observed and expected agreement under the run-specific marginal rates. All reported structured baseline and GEPA runs used N=1076 examples.

Method	Accuracy	Precision	Recall	$F_{1}$	MCC	κ	$\hat{\pi }\left(\text{include}\right)$	Utility
Structured baseline	0.788 ± 0.004	0.791 ± 0.003	0.896 ± 0.006	0.840 ± 0.004	0.539 ± 0.010	0.530 ± 0.009	0.704 ± 0.005	0.847 ± 0.004
GEPA (max_eval=2)	0.776 ± 0.038	0.762 ± 0.052	**0.938**±**0.033**	0.839 ± 0.019	0.518 ± 0.081	0.482 ± 0.106	0.768 ± 0.075	0.850 ± 0.017
GEPA (max_eval=6)	0.786 ± 0.045	0.792 ± 0.072	0.907 ± 0.050	0.842 ± 0.021	0.540 ± 0.099	0.516 ± 0.129	0.719 ± 0.105	0.849 ± 0.016
GEPA (max_eval=12)	**0.797**±**0.023**	0.799 ± 0.043	0.905 ± 0.030	**0.848**±**0.011**	**0.562**±**0.053**	**0.548**±**0.066**	0.706 ± 0.060	**0.855**±**0.007**
GEPA (max_eval=24)	0.780 ± 0.039	**0.811**±**0.071**	0.855 ± 0.050	0.829 ± 0.021	0.529 ± 0.102	0.519 ± 0.107	0.661 ± 0.091	0.832 ± 0.019

**Table C3:** Differences (mean ± sample SD over 5 splits) between each GEPA setting and the structured baseline on the corresponding seed. Positive values indicate higher metric values relative to baseline. All paired comparisons used the corresponding seed and held-out set.

GEPA budget	ΔAccuracy	ΔPrecision	ΔRecall	$\Delta F_{1}$	$\Delta \hat{\pi }\left(\text{include}\right)$	ΔUtility
max_eval=2	−0.012±0.036	−0.028±0.051	+0.042±0.039	−0.001±0.018	+0.064±0.078	+0.003±0.016
max_eval=6	−0.002±0.045	+0.001±0.072	+0.010±0.049	+0.002±0.020	+0.015±0.106	+0.001±0.015
max_eval=12	+0.009±0.020	+0.009±0.041	+0.009±0.034	+0.008±0.008	+0.002±0.061	+0.008±0.005
max_eval=24	−0.008±0.039	+0.021±0.071	−0.041±0.047	−0.011±0.020	−0.043±0.089	−0.015±0.018

**Table D1:** Compact execution environment record for reproducibility. The listed configuration documents the verified local and archived validation runs; it is not a required platform. The tutorial can be run in Google Colab or another compatible Python environment, and users may substitute supported student and reflection models depending on cost, privacy rules, institutional requirements, and availability.

Component	Verified configuration
Verification date	24 April 2026
Operating system	Windows 11; not required for the method
Alternative execution environment	Google Colab or equivalent Python runtime with the listed packages
Python	3.11.15
DSPy	3.2.0
GEPA package	0.0.27
LiteLLM	1.82.6
OpenAI Python SDK	2.32.0
Pandas	3.0.2
scikit-learn	1.8.0
Provider routing convention	LiteLLM model strings with environment-based API credentials
Tutorial / smoke-test student model used in verification	openai/gpt-4.1-mini; replaceable with another supported student model
Tutorial / smoke-test reflection model used in verification	openai/gpt-5-mini; replaceable with another stronger reflection model
Validation student model in archived experiment	openrouter/qwen/qwen-2.5-7b-instruct
Validation reflection model in archived experiment	openai/gpt-5-mini

**Listing E.11:**
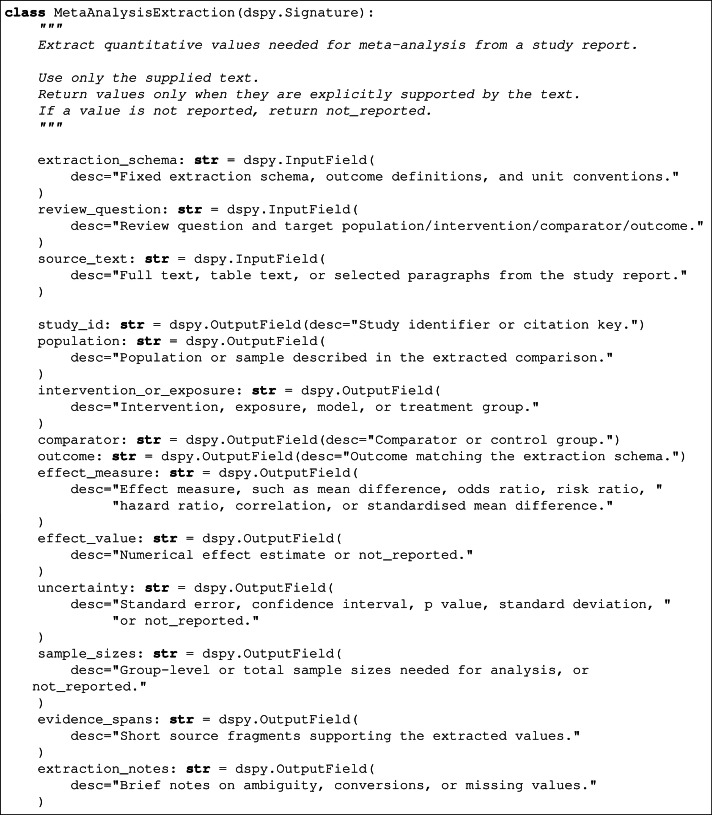
Schematic DSPy signature for meta-analysis data extraction.

**Listing E.12:**
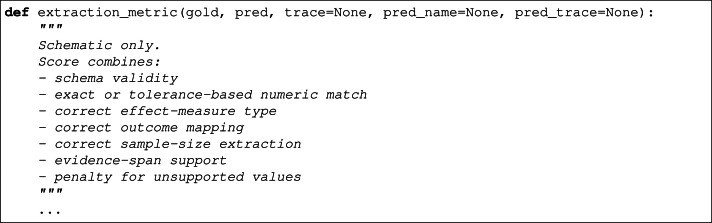
Schematic metric design for extraction calibration.

## Data Availability

The data is on Google Colab

## Appendix A Prompt references and prompt harnesses

The code listings in the article omit the full embedded example records, but the prompt text remains part of the method. The label-only direct-prompt reference documents an ordinary manual-instruction comparator, while the prompt harness records the prompt text that GEPA is allowed to revise. The principal validation baseline is the structured unoptimised DSPy program described in Appendix A. The inclusion and exclusion criteria are not part of the mutable harness; they are supplied as fixed inputs through the criteria field. This prevents the optimiser from changing the review protocol while allowing it to change how the student model interprets and applies that protocol.

This appendix reports the label-only direct-prompt reference, frozen criteria block, structured unoptimised baseline, starting prompt harness, and an excerpt from a compiled harness. These materials allow readers to inspect what was fixed, what was mutable, and what GEPA changed during calibration. This reporting choice matters because two prompt-based LLM workflows may use the same model and nominally the same task criteria, yet still differ in instructions, required output format, uncertainty handling, and post-processing.

### Label-only direct-prompt reference and frozen criteria

Listing A.5 gives the exact label-only direct-prompt reference, and Listing A.6 gives the frozen criteria block. The prompt requested only a single screening label and did not require a structured checks trace. It is therefore reported as a practical reference for ordinary direct prompting, not as the principal comparator for attributing the effect of GEPA calibration. The main validation comparison uses the structured unoptimised DSPy baseline described in Appendix A.

### Structured unoptimised DSPy baseline

The structured unoptimised baseline used the same AbstractScreening signature and Screener module as the GEPA-optimised programs, but was evaluated before calling gepa.compile(). It therefore retained the same input fields, output fields, adapter, student model, temperature, criteria text, label normalisation, and held-out evaluation records as the GEPA conditions. This baseline isolates the effect of GEPA optimisation from the effect of using a structured output contract.

Operationally, the structured baseline is the unoptimised DSPy module produced by student = Screener(). The GEPA condition begins from the same module but passes it to gepa.compile(student, trainset=trainset, valset=valset) before held-out evaluation. This pairing keeps the machine-readable interface constant while allowing GEPA to revise the prompt harness during calibration.

### Starting prompt harness

Listing A.7 shows the mutable starting prompt harness used in the Colab notebook and as the initial GEPA calibration target.

### Example of a compiled harness

The exact compiled text depends on the labelled examples, metric feedback, reflection model, and optimisation budget. A compiled harness often expands the starting prompt by adding guidance for common mistakes, clearer rules for borderline cases, and more explicit instructions for satisfying the fixed output contract. Listing A.8 gives an excerpt from an actual optimised prompt generated during validation. Compared with the starting harness, the compiled harness is more explicit about scope boundaries, clause-level checks, conservative inclusion policy, and the DSPy field structure required by the evaluation pipeline.

## Appendix B Metric-design notes for GEPA optimisation

The main text presents a compact metric because the article body teaches the protocol rather than reproducing every validation check used in the local experiment. This appendix records the richer logic behind the local-testing metric and clarifies which parts are essential, optional, or policy-dependent.

### Why the metric is more than an accuracy check

In DSPy, an optimiser does not discover a universally good prompt. It discovers a prompt that maximises the declared metric. The metric should therefore be understood as the task utility that GEPA is asked to maximise. In screening, this utility is asymmetric: missing a relevant record is usually more harmful than forwarding an irrelevant one to full-text review. The metric therefore gives correct predictions full credit, false negatives zero credit, and false positives partial credit. This scoring tells the optimiser how to trade precision against recall.

### Richer local-testing metric

Listing B.9 gives the expanded local-testing metric used for validation and smoke testing.

### What each component is doing

The expanded metric performs five distinct jobs.

1. It scores label correctness, but in a cost-sensitive way rather than a symmetric exact-match way.
2. It distinguishes false negatives from false positives, so GEPA can prefer prompt variants that preserve recall.
3. It enforces the output schema, because semantically plausible but structurally invalid labels are unusable in an executable DSPy program.
4. It preserves a bounded checks trace, which acts as a compact criteria checklist rather than unrestricted reasoning.
5. It returns case-specific feedback explaining the error, which gives the reflection model more useful revision material than a bare scalar score.

The case block is especially important in reflective optimisation. The metric attaches the truncated criteria, title, and abstract to the feedback. This turns a generic message such as “false negative” into an example-grounded diagnosis. The reflection model can then revise the prompt against the actual failure mode instead of inferring a change from aggregate performance alone.

### Why malformed checks are only partially penalised

The checks field is not the primary prediction target. The primary target is still the final label. The limited penalty on malformed checks therefore expresses a deliberate compromise: semantic correctness remains most important, but the optimiser is discouraged from dropping the structured trace entirely. If the label is already wrong, malformed checks do not materially change the outcome. If the label is correct, however, capping the score prevents GEPA from learning that checklist compliance is optional.

### Interpreting the score correctly

The GEPA metric is a score used during optimisation, not a general performance metric. It can prefer prompts that slightly lower precision if they reduce the risk of missing relevant studies. For this reason, the paper reports conventional held-out metrics separately after optimisation. The optimisation metric defines which prompt variants are favoured during search; the evaluation metrics describe how the resulting program behaves on unseen examples.

The scalar values should be interpreted as a task-utility scale, not as calibrated probabilities. Their order reflects the chosen policy: correct outputs should score highest, false exclusions should be worse than false inclusions in high-recall screening, and malformed outputs should be penalised even when the semantic label is correct. The magnitudes also matter because GEPA compares candidate programs using aggregate metric scores. Raising the false-positive score makes the optimiser more tolerant of over-inclusion and may shift the prompt towards recall; lowering it makes the optimiser more conservative and may favour precision at the cost of missed records. The value 0.4 was used as a moderate partial-credit setting, not as a universal constant. Users should treat these values as policy parameters and should report them with the compiled artefact.

Table B.9 summarises how false-positive scores and checks penalties change the optimisation signal.

### Sensitivity of the false-positive utility parameter

The false-positive score determines how much the metric tolerates false positives compared with false negatives. Table B.10 recomputes the label-level utility from the archived confusion matrices under three plausible false-positive scores. This is a post-hoc illustration only; it does not rerun GEPA under each score. A full sensitivity analysis would repeat optimisation for each candidate utility value because changing the metric can change the selected prompts.

### How this changes in multiclass settings

The binary asymmetry used here does not transfer directly to multiclass screening. In a multiclass problem, each error is simultaneously a false negative for the true class and a false positive for the predicted class. A more appropriate design is then a cost matrix over class pairs. For example, predicting uncertain for a truly includable record may be less severe than predicting exclude, because an uncertain record can still be routed to human review. Multiclass metrics therefore require class-pair-specific scores and feedback rather than a single false-positive branch and a single false-negative branch.

### Practical recommendation

The main article shows the compact metric because it is readable and teaches the core method. For adaptation work, users should expect to iterate on the metric before they iterate on GEPA budget. In most screening applications, metric design is the most direct way to make the optimiser reward the decisions the review process needs, because the optimiser can only learn the screening policy that the metric makes explicit.

## Appendix C Validation configuration and GEPA budget ablation

This appendix reports the validation details that support the concise Method validation section, so readers can reproduce the experiment without turning the main article into a benchmark-style results report.

### Interpreting the budget ablation

Across five repeated splits, the structured baseline achieved an accuracy of 0.788 ± 0.004 and an *F*_1_ of 0.840 ± 0.004, with recall 0.896 ± 0.006 and precision 0.791 ± 0.003 for the include class. The gold include prevalence was approximately 0.621 across all splits. With a small optimisation budget (max_eval=2), GEPA increases recall to 0.938 ± 0.033 while reducing precision to 0.762 ± 0.052 and increasing the predicted include rate to 0.768 ± 0.075. The paired deltas show that this setting primarily reduces false negatives at the cost of additional false positives. Relative to the structured baseline, recall increases by +0.042±0.039, precision decreases by −0.028±0.051, and the predicted include rate increases by +0.064±0.078. This pattern is consistent with the asymmetric metric shaping that penalises false negatives more strongly than false positives.

In this validation setting, max_eval=12 produced the best balance of recall, precision, and workload. It had the highest mean *F*_1_ (0.848 ± 0.011), accuracy (0.797 ± 0.023), and label-level utility (0.855 ± 0.007), with recall 0.905 ± 0.030 and precision 0.799 ± 0.043. Relative to the structured baseline on corresponding seeds, max_eval=12 had $\Delta F_{1}=+0.008\pm 0.008$ and Δutility =+0.008±0.005, while changing the predicted include rate by only +0.002±0.061. MCC and Cohen’s *κ* show the same broad pattern, which is consistent with a modest balanced shift rather than only a large shift in predicted include rate.

Increasing the budget to max_eval=24 produced a non-monotonic outcome. Mean recall (0.855 ± 0.050), *F*_1_ (0.829 ± 0.021), and utility (0.832 ± 0.019) fell below the structured baseline, despite higher mean precision (0.811 ± 0.071). This pattern may indicate instability or overfitting to the small validation set. It shows why optimisation budget should be treated as a controlled hyperparameter rather than a setting that always helps when increased. For abstract screening workflows that prioritise minimising missed relevant studies, max_eval=2 offers the highest recall at the cost of workload. In contrast, max_eval=12 offers the best balance with almost no mean increase in predicted include rate. The lower mean values at max_eval=24 are consistent with over-specialisation under a small validation signal. The validation split contains only 59 examples, so repeated reflective revisions may begin to fit idiosyncratic boundary cases rather than the broader held-out distribution. This does not prove that larger GEPA budgets always overfit. It illustrates a practical constraint: the safe optimisation budget is bounded by the size and diversity of the validation set. In small-label settings, users should treat max_full_evals as a hyperparameter, inspect held-out performance across seeds, and prefer the smallest budget that gives stable held-out behaviour.

### Contextual comparison with SESR-Eval

The SESR-Eval benchmark provides useful context for the size of the observed scores, although its testing protocols are not directly comparable. In SESR-Eval testing, GPT-4.1 mini tended to produce the best results. In the validation setting here, the optimised Study 41 workflow reached a mean accuracy of 0.833 ± 0.008 when optimised with GPT-4.1 mini. SESR-Eval reports an aggregate Study 41 accuracy of 0.78 across all evaluated LLMs in their Table IX, and an average secondary-study accuracy of 0.73 for GPT-4.1 mini across 24 studies in Table VII [18]. These figures should be interpreted only as indicative because the prompt architecture, output requirements, and experimental setup differ. SESR-Eval used a more elaborate screening prompt and Likert-style decision procedure, whereas the present study evaluates a calibrated binary screening workflow designed to focus on reproducibility and prompt optimisation. Also, the present validation places the calibrated 7B-class student model in a similar accuracy range as the published SESR-Eval aggregate figures for Study 41. Direct ranking is not appropriate because the prompts, output formats, decision procedures, and evaluation setups differ. This context matters because the SESR-Eval benchmark was dominated by very large proprietary or open models, including several OpenAI models, Claude 3.7 Sonnet, Llama 4 Maverick (400B), and DeepSeek R1 (671B). Ministral 8B was the only clearly small open model in their comparison set [18].

## Appendix D Execution environment used for local verification

Table D.14 records the environment used for local verification and archived validation, not a required execution environment. The code can run in Google Colab or any compatible Python environment that supports the listed package versions and model-provider configuration. The operating system documents the verified local run only; it is not required by the method. Users may substitute locally hosted or open-weight student models where privacy, cost, or governance requirements favour local inference.

## Appendix E Schematic adaptation for meta-analysis data extraction

This appendix is illustrative only. It is not an empirical validation. It shows how the same calibration pattern can be adapted to a structured extraction task when labelled examples and a scoring metric are available.

In a meta-analysis extraction task, the fixed task rules include the review’s extraction schema, outcome definitions, eligible effect measures, unit rules, and conventions for prioritising adjusted versus unadjusted estimates. The input may be a full text, a table, or a selected collection of paragraphs. The output is a structured extraction record containing the study identifier, population, intervention or exposure, comparator, outcome, effect measure, numerical estimate, uncertainty measure, sample sizes, and evidence spans. Listing E.11 sketches the corresponding DSPy signature.

The extraction metric would differ from the screening metric because the output is a multi-field record rather than a binary label. A compact design is shown in Listing E.12. It would give full credit for correct numeric values within a pre-specified tolerance, partial credit for correct field identification with missing uncertainty, and strong penalties for values that are not supported by the text. It should also penalise missing evidence spans, handle not_reported explicitly when the gold record indicates missingness, and apply higher penalties when the wrong outcome or comparison is extracted because those errors can corrupt the downstream meta-analysis.

The optimiser should not be asked to infer pooled meta-analytic results. It should calibrate extraction of study-level values only. Statistical pooling remains a downstream analysis step.

## Footnotes

1 MethodsX_GEPA_Tutorial.ipynb accessible at https://colab.research.google.com/drive/1gY_XzQ1h1EsK18bU136t2euiNWlo7sQC?usp=sharing

2 API credentials must be supplied through environment configuration as no key is stored in the notebook or manuscript code.

3 The validation experiments used the expanded version in Appendix B. It adds case-grounded feedback and stricter field-format instructions while preserving the same label-level asymmetric scoring. The supplementary validation code also includes the helper utilities normalize_label() and _checks_format_ok(). Listing B.10 shows them because label normalisation and trace validation are part of the executable evaluation contract.

4 The larger validation at the end of the notebook below evaluates the protocol on SESR-Eval records

5 The validation was conducted in a separate experimental workflow rather than in the companion Colab tutorial notebook. The Colab notebook is an executable teaching artefact that demonstrates the code path, compilation, saving, reloading, and adaptation steps on a small example.
